# Spiers Memorial Lecture: Lithium air batteries – tracking function and failure†

**DOI:** 10.1039/d3fd00154g

**Published:** 2023-12-04

**Authors:** Jana B. Fritzke, James H. J. Ellison, Laurence Brazel, Gabriela Horwitz, Svetlana Menkin, Clare P. Grey

**Affiliations:** a Yusuf Hamied Department of Chemistry, University of Cambridge Cambridge UK cpg27@cam.ac.uk sm2383@cam.ac.uk

## Abstract

The lithium–air battery (LAB) is arguably the battery with the highest energy density, but also a battery with significant challenges to be overcome before it can be used commercially in practical devices. Here, we discuss experimental approaches developed by some of the authors to understand the function and failure of lithium–oxygen batteries. For example, experiments in which nuclear magnetic resonance (NMR) spectroscopy was used to quantify dissolved oxygen concentrations and diffusivity are described. ^17^O magic angle spinning (MAS) NMR spectra of electrodes extracted from batteries at different states of charge (SOC) allowed the electrolyte decomposition products at each stage to be determined. For instance, the formation of Li_2_CO_3_ and LiOH in a dimethoxyethane (DME) solvent and their subsequent removal on charging was followed. Redox mediators have been used to chemically reduce oxygen or to chemically oxidise Li_2_O_2_ in order to prevent electrode clogging by insulating compounds, which leads to lower capacities and rapid degradation; the studies of these mediators represent an area where NMR and electron paramagnetic resonance (EPR) studies could play a role in unravelling reaction mechanisms. Finally, recently developed coupled *in situ* NMR and electrochemical impedance spectroscopy (EIS) are used to characterise the charge transport mechanism in lithium symmetric cells and to distinguish between electronic and ionic transport, demonstrating the formation of transient (soft) shorts in common lithium–oxygen electrolytes. More stable solid electrolyte interphases are formed under an oxygen atmosphere, which helps stabilise the lithium anode on cycling.

## Abstract 2

Here, we discuss experimental approaches developed by some of the authors to understand the function and failure of lithium–oxygen batteries.

## Graphical abstract

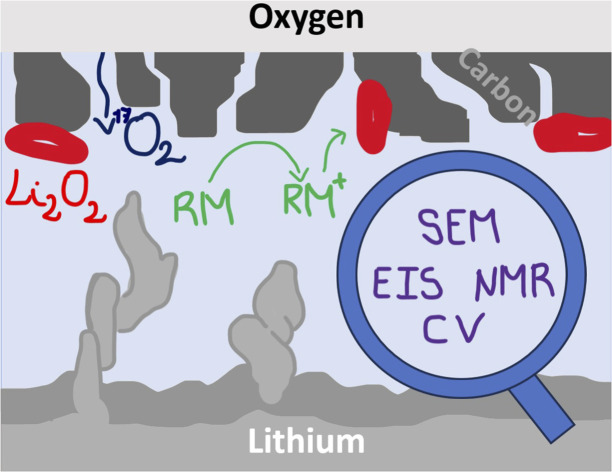

## 1 Introduction

The lithium–air battery (LAB) promises up to ten times higher theoretical energy density than today’s lithium-ion batteries, uses abundant, free, non-toxic, and light-weight oxygen as the active material, and is easier to recycle. However, in practice, its capacity is much lower and its rate performance is poor. Cycling is associated with high overpotentials, resulting in low energy efficiency, and the accompanying parasitic reactions result in short cycle lives. Lithium is often used at the anode, bringing with it its own series of problems, including poor coulombic efficiency (due to a series of parasitic reactions that lead to the formation of the solid electrolyte interphase (SEI) and dendrite formation). Suitable membranes are likely needed to prevent crossover of oxygen and the degradation products formed at both electrodes and, at least in principle, to help mitigate dendrite issues. Furthermore, these challenges are for cells that use oxygen gas, strictly lithium–oxygen batteries (LOBs), and the use of air brings with it additional problems, primarily due to the presence of carbon dioxide.

The various challenges of LABs have been widely discussed and reviewed by us and others.^1,2^ For example, some of us in this discussion series (Ellison *et al.*, https://doi.org/10.1039/D3FD00091E) proposed that to construct a practical high-energy-density LAB that would operate with a moderate cycle rate, the air would need to be compressed to around 20 bar and a highly porous (>90%) carbon electrode approximately 100 μm thick would be required. The electrolyte would also need to have a high boiling point (*ca.* 250 °C) to prevent excess evaporation and have favourable oxygen transport properties, enabled *via*, for example, the solvent molecules and/or salts containing apolar fluorinated or alkyl regions. Possible approaches to easing these requirements include the use of hierarchical porous structures, pumping the electrolyte though the cell or further increasing the pressure of the overhead gas in the cell. Some of these approaches present significant engineering challenges to realize and all bring with them associated cost and/or mass to the cell.

This paper is restricted to a brief discussion of some of the characterization approaches used to understand function and failure in lithium oxygen batteries. A wide variety of techniques have been use to study the batteries, including Raman spectroscopy to study discharge products,^3^ X-ray photoelectron spectroscopy (XPS) to study the composition of the lithium SEI,^4^ and X-ray diffraction (XRD)^5^ to study the crystalline discharge products, along with others, many of which are discussed in existing reviews.^1,2^ Thus, we focus largely on reviewing a number of approaches developed by some of the authors, which generally (but not exclusively) involve the use of NMR spectroscopy, and then end by presenting new results on the characterization of lithium-metal soft shorts in these systems using both EIS and *operando* NMR.

### 1.1 Oxygen solubility

Two factors controlling both the rate and also the distribution of reaction products over the porous carbon electrode are the oxygen solubility and diffusion. With poor solubility and diffusivity, the reactions to form the insoluble lithium peroxides occur predominantly near the electrolyte–gas interface. This leads to clogging of the carbon pores near this interface and early cell death before all of the available pores have been filled with reaction products.

**Fig. 1:**
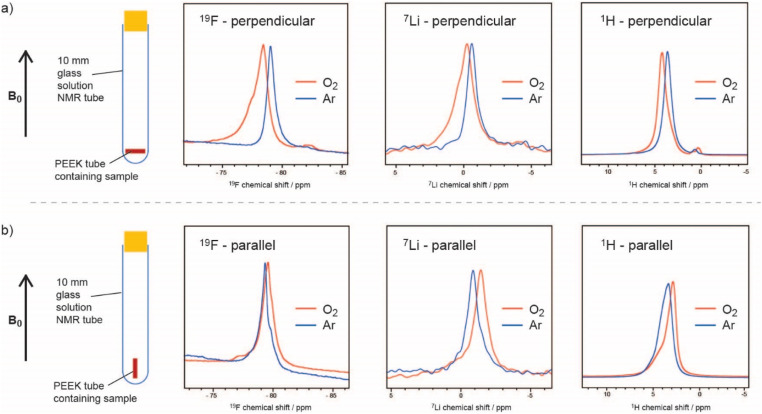
^19^F, ^7^Li, and ^1^H NMR spectra of a 0.25 M LiTFSI in diglyme electrolyte, where the sample is oriented (a) perpendicular and (b) parallel to the applied magnetic field, before (blue) and after (orange) O_2_ saturation, showing the noticeable BMS shifts due to dissolved paramagnetic oxygen. The –CH_3_ peak of diglyme is shown for the ^1^H spectra. Reproduced with permission from ref. 8 under the Creative Commons license CC-BY 4.0, Copyright 2023.

Traditional methods to measure oxygen solubility include measuring changes in pressure/volume on exposure of an electrolyte to a headspace of oxygen.^6,7^ We have explored the use of NMR approaches since the NMR method can, in principle, separate between bulk uptake in the electrolyte and interactions of oxygen with specific molecules and ions.^8^ In Fig. 1, the small but noticeable bulk magnetic susceptibility (BMS) shifts seen due to dissolved paramagnetic (*S* = 1) oxygen molecules are confirmed by studying small tubes filled with electrolyte and gas as a function of orientation with respect to the magnetic field. A shift to negative frequencies for a sample oriented parallel to the field indicates that the bulk (here a liquid) is paramagnetic. The difference in shifts between the perpendicular and parallel orientations can be directly converted into its volume susceptibility, which in turn can be used to calculate the quantity of dissolved oxygen. This simple approach was shown to yield oxygen concentrations that were similar to those measured by traditional methods. The method could be modified to track oxygen uptake by the solution in real time, from which diffusion coefficients could be extracted. In addition to the BMS shifts, small hyperfine shifts were seen, reflecting direct interactions between the solvent/ion atoms and the oxygen molecules. The larger ^19^F shifts were interpreted in terms of preferential solvation of the dissolved O_2_ by the F atoms in the bis(trifluoromethane)sulfonimide (TFSI^−^) salt anion, particularly in glyme electrolytes. Of the electrolytes studied, the highest O_2_ solubility and diffusivity and largest ^19^F hyperfine shifts were seen when using the lower-molecular-weight glyme as a solvent. The dimethylsulfoxide (DMSO)-based electrolytes had lower O_2_ solubilities, with both the ^19^F and ^7^Li NMR spectra showing hyperfine interactions, indicating that O_2_ interacts with both the Li^+^ ions and F atoms of the TFSI anions. This was consistent with the weaker DMSO–Li^+^, in comparison to the glyme–Li^+^ interactions, the freer Li^+^ ions being more available to bind to O_2_. By contrast, in the glymes, the TFSI anions are freer and more able to bind to oxygen.

The effect of the paramagnetic oxygen molecules on the ^1^H, ^19^F and ^7^Li spin lattice relaxation times was also explored and the approach was then used in preliminary *in situ* NMR experiments to track changes in dissolved oxygen concentrations.

### 1.2 Characterising degradation with NMR spectroscopy

NMR has been used extensively to track Li_2_O_2_ formation and track degradation of the electrolyte. The only NMR-active nucleus for oxygen is ^17^O, which has a very low natural abundance (0.038%) and a large quadrupole moment, resulting in both weak and typically broad signals. Enrichment is expensive so experiments cannot cannot be performed with large volumes of oxygen gas. Early studies used a head-space filled with 20–25% ^17^O-enriched oxygen and compared the ^17^O MAS NMR spectra of electrodes extracted from batteries at different states of charge (SOC) to the spectra simulated or obtained directly for different degradation components. ^1^H NMR spectra have also been used to monitor electrolyte degradation components.

**Fig. 2:**
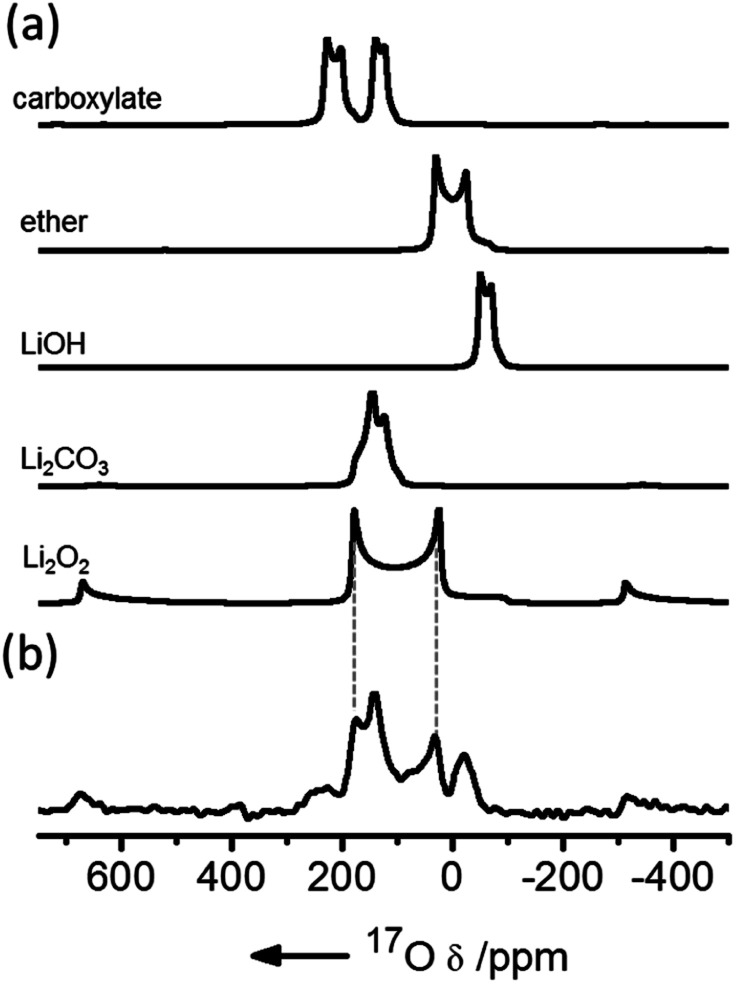
(a) Simulated ^17^O magic angle spinning (MAS) NMR spectra of possible discharge products compared with (b) the experimental spectrum of a cathode extracted from a cell prepared with a 1 M bis(trifluoromethane)sulfonimide lithium (LiTFSI) electrolyte in dimethoxyethane (DME) solution, after discharge, acquired at a field of 21.1 T and fast (60 kHz) MAS. Reproduced with permission from ref. 9 under the Creative Commons license CC-BY, Copyright 2013.

This early ^17^O NMR work showed that the major breakdown products in a DME solvent were Li_2_CO_3_ and LiOH, with a smaller amount of HCO_2_Li being formed (Fig. 2).^9^ With *ex situ* measurements, it was found that LiOH was partially removed at voltages lower than 4.5 V, with Li_2_CO_3_ being removed just above this voltage. In addition, ^13^C homonuclear correlation experiments showed that at least some Li_2_CO_3_ that was formed was in direct contact with the carbon electrode, as has been observed in other works using isotopic enrichment of the carbon.^10^ Ongoing work in our group has focussed on the development of metrology to allow these measurements to be performed *operando*; simultaneous *operando* solution NMR measurements also allow water formation, for example, to be tracked, which on discharge appears to be linked to the presence of singlet oxygen.^11^

### 1.3 Redox mediators

Redox mediators have been used both on charge and discharge to perform chemical oxidation and reduction of Li_2_O_2_ and oxygen, respectively, the electrochemical processes occurring *via* the use of dissolved redox-active molecules/ions that are either oxidized or reduced at the current collectors. These mediators help solve one major problem, namely that the reaction product, Li_2_O_2_, is an insulator and once formed on the electrode on discharge, it effectively blocks further electrochemical reactions on the surface area that it covers. On charge, direct oxidation of Li_2_O_2_ at the carbon–Li_2_O_2_ interface is possible but, without a mediator, the charge overpotentials increase dramatically as electrical contact is lost as the Li_2_O_2_ is removed. The resulting high overpotentials drive accompanying parasitic reactions, such as the oxidation of the solvent molecules, which leads to the accumulation of inactive products.

**Fig. 3:**
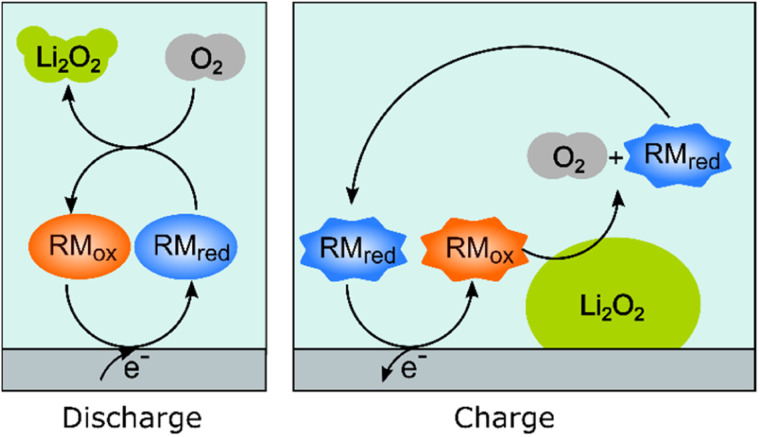
Schematic representation of how redox-active molecules can be used to aid the discharge and charge processes by enabling oxygen reduction and oxygen evolution reactions (ORR and OER, respectively) to occur chemically in solution as opposed to electrochemically on the surface of the electrode.

Fig. 3 schematically illustrates how redox mediators operate. On discharge, the redox mediators react directly with dissolved oxygen, promoting solution-based reaction processes that allow large Li_2_O_2_ crystals to be obtained throughout the porous structure, as opposed to thin film coatings that quickly passivate the surface. For example, by using DBBQ (2,5-di-*tert*-butyl-1,4-benzoquinone) the capacity was increased up to 80 times.^12^ On charge, redox mediators can chemically react at the Li_2_O_2_ particles’ surfaces, significantly lowering overpotentials and improving the energy efficiency, as well as reducing degradation.

Redox mediators must obey certain requirements: clearly, their reduction potential must be below that of the OER to be active during discharge, while the opposite is required for charge, so that the reactions are thermodynamically spontaneous. They must be soluble in the desired solvent and stable under the aggressive conditions of the Li–O_2_ battery. Other parameters also affect their performance. For example, to maximize the energy efficiency of the battery, their reduction potential needs to be close to that of the ORR, and their electron transfer kinetics at the electrode must be fast, to minimize overpotentials. The catalytic kinetics, *i.e.*, the reaction rates between the chosen redox mediator and the target species (either O_2_ or Li_2_O_2_), also need to be fast, and work to understand what determines these reaction rates is an active area of research in the field.^13,14^ Less attention has been paid to the kinetics of discharge redox mediators, however, significant increases in overpotentials and decreases in capacity have been observed when using these mediators under rates closer to practical values, and more work is needed to understand and reduce this phenomenon.^12,15,16^

**Fig. 4:**
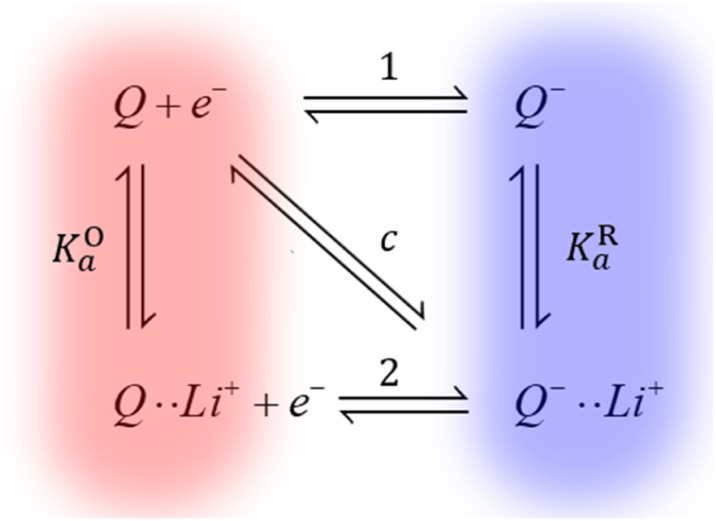
Simplified diagram of the different mechanisms DBBQ, depicted as “*Q*”, can undergo in Li^+^-containing DMSO and glyme solutions. Vertical reactions correspond to chemical equilibria of ionic pairing between quinone species and Li^+^ ions; horizontal reactions correspond to electron transfer reactions. Pathways 1 and 2 correspond to stepwise mechanisms, while pathway *c* denotes the possibility of a concerted reaction where electron and ionic transfer occur simultaneously. *K*^O^_a_ and *K*^R^_a_ denote the association constants of the ion pairing reactions.^17^

We have recently shown how Li^+^ ion concentration can be used as a tuning knob for the electron transfer kinetics of the discharge redox mediator DBBQ.^17^ For example, this redox mediator shows a maximum in its standard electron transfer constant, *k*^0^, at a LiTFSI concentration of around 0.25 M in DMSO-based solutions. We have explained the kinetic effects in the context of a proposed coupled ion-electron transfer theoretical framework, which can be easily extended to other redox mediators (Fig. 4). Overall, in work to be published, we have explained how the interaction of quinones with Li^+^ ions affects the reduction mechanism, and the role of other parallel equilibria and solvation effects. This gives us predictive power to rationally design better and more efficient redox mediators, with better rate performance.

Another challenge when it comes to redox mediators is their cross-over to the anode. Charge redox mediators are susceptible to being reduced by the lithium, resulting in a reduction in coulombic efficiency, and all redox mediators can be susceptible to degradation at the anode’s surface, limiting their cycle life. In a practical Li–O_2_ battery, solutions such as permeable and selective membranes will need to be implemented to avoid their cross-over.^18–20^

The mechanistic changes caused by addition of redox mediators could be studied by using EPR and NMR: EPR has previously been used to study superoxide lifetime, which, along with diffusion, can be linked to lithium-peroxide discharge-product size.^21^ These studies can, however, be challenging due to the short superoxide lifetime in the presence of Li^+^. Some redox mediators are radicals in either their oxidised or reduced states. A good example is TEMPO ((2,2,6,6-tetramethylpiperidin-1-yl)oxyl), and its concentration can be followed *operando via* EPR.^8^

### 1.4 The use of lithium metal as an anode

Lithium metal typically plates in liquid organic electrolytes, although this does not necessarily hold true if a ceramic membrane is used to separate the anode from the oxygen (cathode) side. In a liquid cell, however, the battery electrolyte’s reduction potential is higher than that for alkali-metal ion reduction, and an SEI is formed upon the contact of the metal with the electrolyte by precipitation of electrolyte reduction and degradation products. The Li morphology and plating efficiency have been shown to correlate with the transport properties and formation kinetics of the SEI, which influences the current distribution over the electrode surface.^22^

During the cycling of a metal battery, the SEI breaks down and is repaired periodically due to the significant volume changes of the Li anode. Hence, new SEI forms constantly, while excess SEI and dead lithium (lithium that is electrically disconnected from the current collector and cannot cycle) accumulates, and electrolyte is consumed. While in lithium metal batteries, the SEI’s composition and structure are determined mainly by the liquid electrolyte composition, in Li–air cells, it is also affected by the composition of the gas headspace in the cell, cross-over of gases, electrolyte decomposition products that result from the reactive oxygen species and potentially redox mediators.

**Fig. 5:**
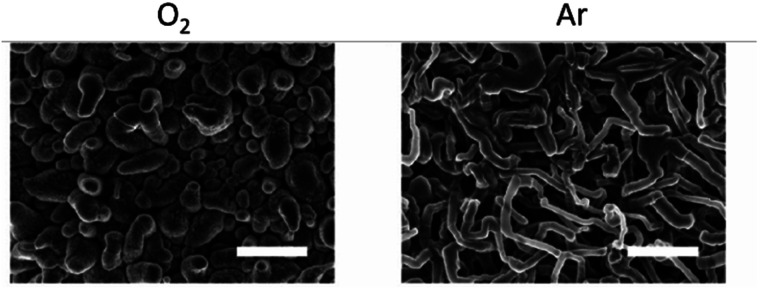
Effect of O_2_ on Li nucleation. SEM images after the first Li plating in Ar (right) and O_2_ (left). Scale bar = 10 μm. Reproduced with permission from ref. 4 under the Creative Commons license CC-BY, Copyright 2020.

In our previous studies, Wang *et al.* showed that in Li–air batteries, dissolved gas can cross over from the air electrode to the Li-metal anode and affect the solid-electrolyte interphase (SEI) formation.^4^ The presence of O_2_ significantly improved the lithium cyclability and decreased Li loss. However, the SEI resistivity and plating overpotentials increased. They demonstrated improved coulombic efficiencies when cycling lithium in the presence of O_2_, O_2_ helping to form a more homogeneous LiOH-containing SEI layer on the copper (Cu) substrate and Li-metal surface, enabling more uniform Li nucleation and improving subsequent plating–stripping efficiencies.^4^ In the presence of Ar, a more heterogeneous SEI was formed, which resulted in nonuniform plating and dendrite growth. Encouragingly, the best performance was obtained for O_2_/N_2_ ratios, close to those found in air (Fig. 5).^4^

The overpotential (*η*_t_) that develops during plating and stripping a metal originates from the ohmic resistance (*η*_ir_) of the system, and the charge-transfer resistance at the electrode surface that includes the activation energy barrier of metal nucleation (*η*_ac_). The SEI introduces an additional overpotential (*η*_SEI_) due to hindered Li^+^ transport through the interphase layer and potentially, the ion concentration polarisation at the SEI-electrolyte interphase. The overall overpotential in these systems can be summarised as:

1 *η*_t_ = *η*_ir_ + *η*_SEI_ + *η*_ac_

Prolonged galvanostatic cycling of symmetric cells has been regarded as a key metric indicating the viability of a particular metal anode–electrolyte system. The voltage traces, *i.e.*, changes in overpotential during plating and stripping, yield information on the nucleation and growth of metal microstructures.^23^ A sharp nucleation peak at the beginning of plating has often been associated with dendritic Li morphology, and a rectangular-shaped voltage profile with a minimal overpotential is often reported as ideal.^24^ However, some of us showed in this Discussion series (https://doi.org/10.1039/D3FD00101F) that this rectangular shape is characteristic of a soft-shorted cell, where electronic transport through the contact of the tip of a dendrite and the counter electrode competes with ionic transport through the SEI and electrolyte. This regime is distinct from a conventional short circuit because, in the soft-short case, the resistances through the ionic and electronic paths are comparable, leading to a mixed conduction regime (https://doi.org/10.1039/D3FD00101F).

**Fig. 6:**
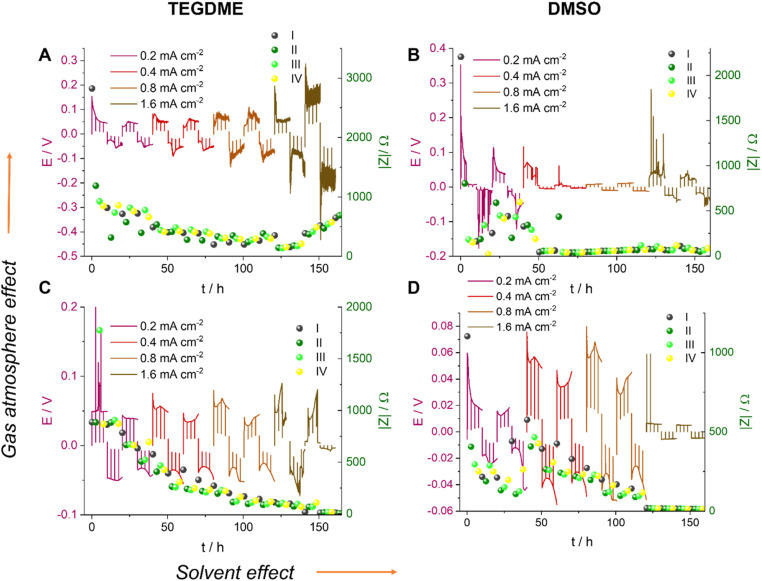
The voltage traces of galvanostatic cycling for 1 M LiTFSI electrolyte in TEGDME (A and C) and DMSO (B and D), in Ar (A and B) and O_2_ (C and D) atmospheres with currents of 0.2, 0.4, 0.8 and 1.6 mA cm^−2^. The I–IV labels represent different stages in the cycle: (I) nucleation, (II) surface area growth, (III) current direction switch, and (IV) plating on the opposite electrode while stripping the dendrites and stripping bulk lithium, respectively.

We now, in the section Results and discussion, use our recently developed methodology, where we combine *in situ* NMR and electrochemical impedance spectroscopy (EIS), to investigate the formation of short circuits in lithium symmetric cells using electrolytes and gas environments that are relevant for LOBs. Comparing the most commonly used electrolytes in argon and oxygen environments, we demonstrate that while the tetraethylene glycol dimethyl ether (TEGDME)-based 1 M LiTFSI electrolyte better withstands polarisation, potentially due to a higher critical current density (CCD), it gives rise to more noisy voltage traces, which could potentially indicate dead lithium accumulation or soft shorts. In contrast, the DMSO-based electrolyte suffers from more significant polarisation and forms soft shorts at lower current densities (Fig. 6). This trend is especially pronounced when the headspace of the cell is filled with argon. *Operando* Li-NMR spectroscopy is then used to help understand the difference: by following the ^7^Li signals corresponding to bulk lithium metal and lithium microstructures, the morphology of the plating/stripping is correlated to the electrochemical behaviour.

## 2 Results and discussion

Fig. 6 shows the voltage traces of galvanostatic cycling for 1 M LiTFSI electrolyte in TEGDME and DMSO, in either an Ar or O_2_ atmosphere. Symmetric cells were cycled with a galvanostatic protocol at 0.2–1.6 mA cm^−2^ to study lithium-metal plating and the rate capability of the cell. The current density was increased every two cycles while the time between the current direction switches remained constant. Although typically the amount of cycled lithium (charge) is kept constant, we kept the time constant here and instead increased the charge *via* the current density so as to increase the plating-related cell degradation and demonstrate soft shorts. Note that the maximum plated lithium capacity of 4 mA h cm^−2^ is typical for commercial batteries. A longer total cycling time would promote degradation due to electrolyte depletion, evaporation, and corrosion, which would potentially mask the signature of transient short circuits. The charge transport across the cell during cycling was measured with *in situ* EIS. The measurement was conducted in galvanostatic mode and in a narrow frequency range (500–0.8 Hz). The narrower range minimises the effect of the EIS measurement on the voltage trace, since the spectrum acquisition lasts only nine seconds; low frequencies, which might result in plating and stripping during the EIS measurement, are avoided. Indeed, the prolonged galvanostatic experiments resulted in typical smooth voltage traces despite the disruptions due to the galvanostatic EIS (GEIS) (which resulted in the spikes seen in the voltage traces; see Fig. 6). In work in this Discussion series (https://doi.org/10.1039/D3FD00101F), in Fig. S2 of the ESI,† we compare the plots of impedance intensity *vs.* time in the 500–0.8 Hz range of measured frequencies. The impedance traces show similar trends and the measurements at 9 Hz were chosen as representative GEIS measurements. Using a single frequency allows us to correlate the impedance intensity to the voltage (overpotential) profile.

The EIS measurements were taken every 2.5 hours immediately after the plating was paused. Isolated EIS measurements around the open-circuit voltage (OCV) eliminate the effect of the redox reaction’s activation overpotential and ion migration, allowing the measurement of pure charge transport across the cell.

**Fig. 7:**
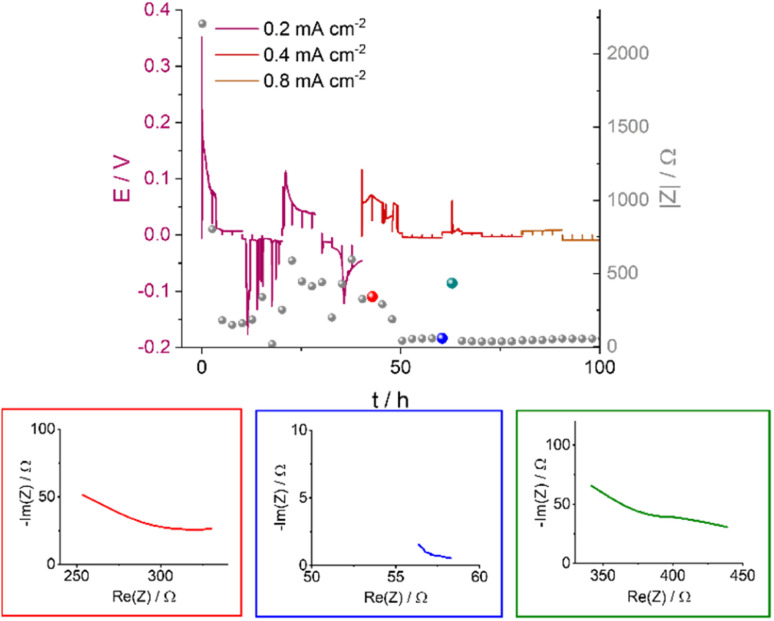
The voltage traces of galvanostatic cycling for 1 M LiTFSI in DMSO in an Ar atmosphere with currents of 0.2, 0.4 and 0.8 mA cm^−2^ (magnification of Fig. 6B) and the corresponding impedance magnitude |*Z*| at 9 Hz (grey balls); select Nyquist plots are shown for impedance measurements made at specific time points, illustrated by the red, blue and green balls. The red, blue and green plots correspond to before, during, and after soft shorting occurs.

Soft shorts are generally seen *via* an overpotential drop and fluctuations in the impedance magnitude. The DMSO-based electrolyte in an Ar headspace (Fig. 6B) is associated in the first cycle at 0.2 mA cm^−2^ with noisy voltage traces and impedance fluctuations, which we assign to the onset of recoverable soft shorts (named here type A); these remain during cycles 3 to 6 (hours 50 to 125). A magnification of Fig. 6B is shown in Fig. 7, along with selected Nyquist plots taken at key stages of cycling. After the first two cycles, the current density was increased to 0.4 mA cm^−2^ and the voltage trace shape became rectangular, the impedance magnitude abruptly dropping sixfold (Fig. 7). These are again indicative soft shorts, since such an abrupt impedance drop cannot be explained solely by surface area growth (since these soft shorts have a different impedance and voltage trace, we refer to these as type B soft shorts). Hence, the critical current density for these cells is 0.4 mA cm^−2^.

The first highlighted GEIS measurement in Fig. 7 (red) is taken just after the completion of the second cycle at 0.2 mA cm^−2^, and while the total magnitude of the impedance is lower compared to the pre-cycling measurement (approximately 350 Ω *vs.* 2000 Ω, respectively), it is in the same order of magnitude as impedance after lithium nucleation (about 750 Ω). Hence, it is likely that the impedance decline here is due to surface area (SA) growth during dendrite formation. The Nyquist plot (red EIS trace, Fig. 7) consists of at least one semi-circle with a maximum at a higher frequency than 500 Hz (the curve cannot be fitted due to the limited frequency range used). The next impedance curve (blue) was measured after a complete cycle at 0.4 mA cm^−2^, where a significant drop in the overpotential was observed. The impedance showed a sixfold drop and at least one semi-circle with a maximum of 30 Hz. The variation of the maximal frequency is an indication of a change in the charge transport mechanism. We attribute this change to the soft-short formation. The third impedance measurement magnitude is approximately seven times higher (Fig. 7, green), and the Nyquist plot is closer in shape to before the soft short and has at least two semi-circles: a high (500 Hz) and a low (30 Hz) frequency. The recurrence of the high-frequency semi-circle indicates the soft shorts’ recoverability. While type A soft shorts are mainly indicated *via* a noisy profile, type B soft shorts are indicated *via* a rectangular voltage profile and low overpotential, which can also be misinterpreted as an indication of good performance of the cell.

### 2.1 SEM

**Fig. 8:**
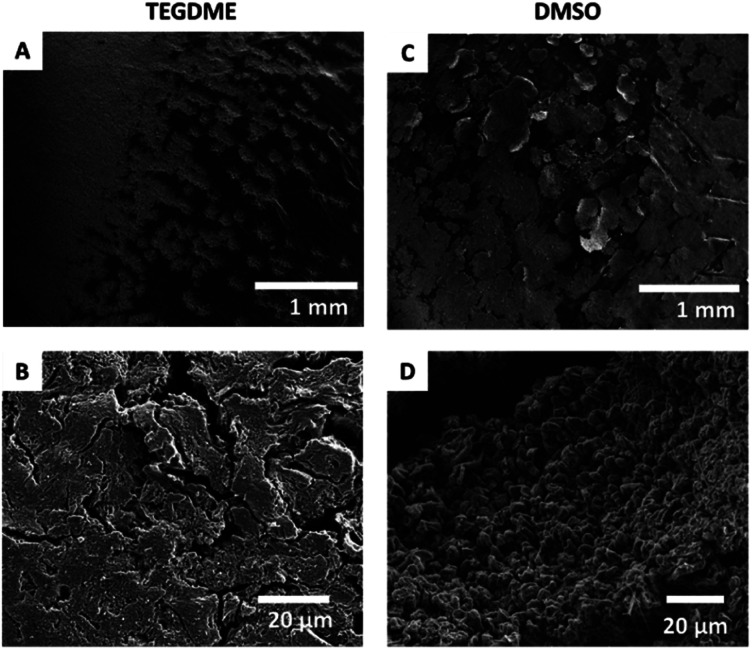
SEM images of the microstructures formed on applying a 1.0 mA cm^−2^ constant current in TEGDME (A and B) and DMSO (C and D) at two different magnifications.

The effect of the solvent on the resulting Li-metal morphology was studied by scanning electron microscopy (SEM). Li was plated after a resting time of 30 hours at a current density of 1 mA cm^−2^ for 3 hours (Fig. 8A–D). A more porous dendritic Li morphology was seen when using TEGDME as the solvent, with a “flower-like” distribution of Li on the electrode (Fig. 8A and B). While the non-dendritic morphology is encouraging, loosely attached Li metal sheets can be observed, that might result in the loss of significant part of active material. In DMSO, by contrast, the Li metal is more flake-like and more evenly distributed on the electrode surface. The microstructures are denser and do not grow away or delaminate from the electrode surface.

### 2.2 *Operando* NMR spectroscopy

**Fig. 9:**
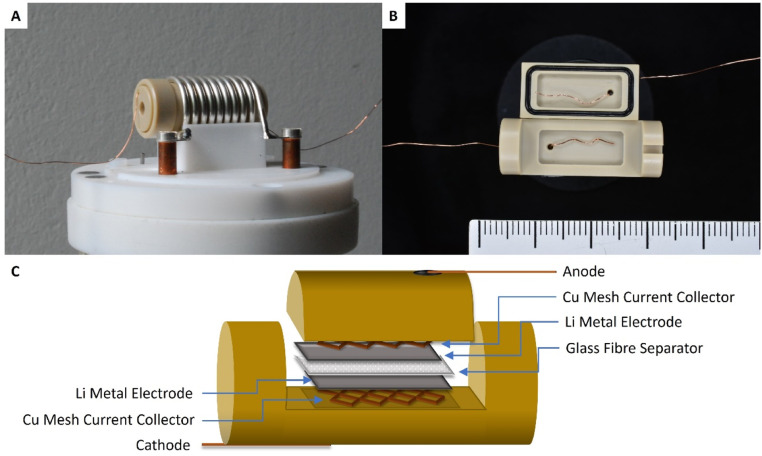
(A) An *in situ* capsule cell placed in the solenoid coil that sits inside of the *in situ* NMR probe. (B) An opened capsule cell with Cu wire as the current collector, also showing the Viton® rubber O-ring seals that help maintain the airtight cell, and a cm scale-bar. (C) Scheme of the symmetrical Li–Li cell stack inside of the capsule cell, showing the two Li-metal electrodes, the glass fibre separator soaked with the electrolyte and the Cu meshes that improve the contact between the wire and the electrode.

All **O*perando* NMR experiments were carried out in capsule cells made out of PEEK (polyether ether ketone) with Viton® rubber O-rings, as shown in Fig. 9 and discussed extensively elsewhere.^25^ A modified cell design^11^ was used for cells with an oxygen gas headspace. The ^7^Li NMR spectrum of this symmetrical Li cell shows two distinct signal regions, the diamagnetic region around 0 ppm and the Knight-shifted region at *ca.* 250 ppm. The diamagnetic peaks can be assigned to Li^+^-ions in the electrolyte and in the SEI in different environments and are not further investigated in this study. The shifts outside the diamagnetic region are assigned to Li metal inside the cell and undergo a change during electrochemical plating. The signal increases in intensity and broadens towards higher shifts. This change in the peak shape and intensity can be explained by two effects: the bulk magnetic susceptibility (BMS) and the skin depth effects. The radio frequency (rf) used to excite the Li-metal signal is only able to penetrate the Li-metal electrodes to a certain depth (*ca.* 12 μm) – the skin depth effect – and hence the intensity of the signal is affected.^22^ Therefore, the detected intensity of the metal signal is sensitive to the surface area and not the volume. Since the diameter of the dendrites is smaller than the skin depth, the increase in Li-metal signal should correlate directly with the mass of plated Li. The BMS shift is caused by the local fields induced by the Li metal, which shows temperature-independent paramagnetism, and can be used to differentiate the bulk metal and the plated dendrites. The plated Li metal and dendrites, referred to as microstructured Li metal in the following description, show a higher shift than the bulk metal of the electrodes of *ca.* 265 to 275 ppm. Hence, as performed by Bhattacharyya *et al.*,^26^ a decomposition of the metal peak was applied to quantify the microstructure formation.

#### 2.2.1 Li-metal plating in TEGDME

**Fig. 10:**
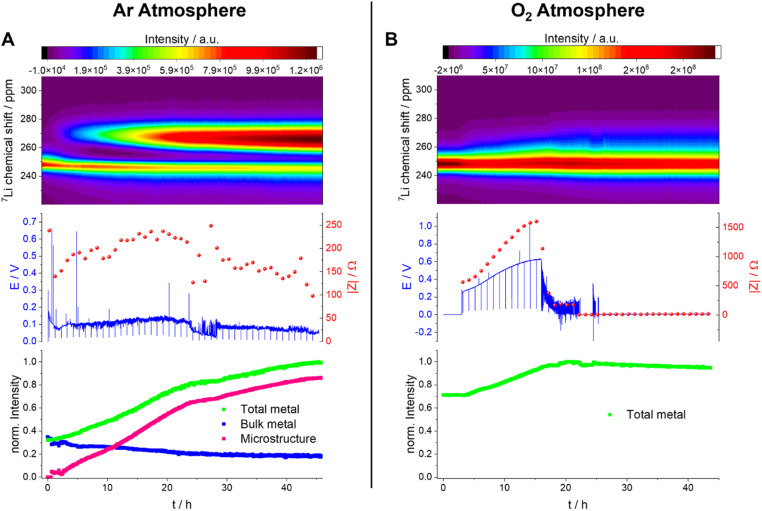
*Operando* NMR spectroscopic measurements of Li-metal plating in symmetrical cells with 1 M LiTFSI in TEGDME under Ar (A) and O_2_ (B) atmospheres. Spectra are plotted for the metal signals only in a shift range between 220 and 310 ppm (top) with the corresponding voltage profile and the impedance magnitude |*Z*| at 9 Hz (middle) and normalised intensity of the metal signal (bottom).

The voltage and impedance of the galvanostatic plating in TEGDME under an Ar atmosphere show a noisy voltage profile with a relatively low overpotential of *ca.* 0.2 V (Fig. 10A), indicating a less stable SEI and potentially the formation of type A soft shorts (Fig. S9 in the ESI†). The impedance of the cell increases in the first half of the experiment, which can be explained by the formation of an SEI on the surface of the anode microstructure. Afterwards, the impedance decreases, with an additional drop after 25 hours that we assign to a soft-short-circuit-based degradation mechanism (Fig. S9†). The measured Nyquist plots (Fig. S9B†) have a typical shape for soft-shorted cells without distinguishable semi-circles (Fig. 7, S1, S2 and S4†).

The plot of the total Li-metal intensity *vs.* time shows that the surface area of the electrodes increases right from the beginning of the plating (Fig. 10A). This intensity increase is due to the appearance of a second peak, in addition to the bulk Li signal, which is assigned to the microstructural metal. The microstructure peak is associated with a high BMS shift, consistent with a dendritic/porous Li-metal structure that grows away from the bulk metal surface (Fig. 10A), in agreement with the Li microstructure seen in the SEM images (Fig. 8). The bulk-metal peak decreases slightly in intensity, which can be explained by the formation of microstructures on the surface that prevent the rf from penetrating the Li-metal electrode to the same depth as at the beginning of the experiment. After 25 hours, there is a noticeable change in the rate of intensity growth of the microstructure peak, with the peak growing much more slowly, indicating that the rate of dendrite formation has reduced significantly and is no longer tracking the current flow. This reduction in the rate of plating is correlated with a drop and more erratic behaviour in the impedance. Both observations are consistent with a soft-short degradation mechanism, the soft short allowing current to flow through the short (electronic transport), and reducing the rate of plating (*via* ionic transport). This soft short was not permanent/able to carry all the current, because plating still continues, albeit at a lower rate, as seen by the further increase in the microstructure and the total-metal peak. This suggests that the short circuits can easily be broken as a result of chemical corrosion or mechanical breakage. Chemical corrosion involving reactions between Li metal and the electrolyte will be particularly pronounced in these shorts, which can carry high currents, resulting in pronounced local heating.

A very different behaviour is seen when measuring a symmetric Li cell in an O_2_ environment (Fig. 10B). After a total of five hours resting time (only hours 3–5 are plotted), performed to enable the diffusion of the O_2_ into the electrolytes, the plating starts with a smooth voltage profile. This same behaviour was seen in Swagelok cells, where an improvement of the cycling behaviour in the presence of O_2_ was clearly detectable (Fig. 6). The overpotential and the impedance increase in the first 16 hours indicate the formation of a thicker SEI and/or an SEI with poorer Li^+^ transport (Fig. 10B and S8†). In the *operando* NMR cell, however, no distinct microstructure peak was observed, and instead a broadening of the main metal peak, and growth in its intensity, is seen, which is characteristic of the formation of more dense microstructural Li metal on the surface of the bulk Li-metal electrode.

After 16 hours, the voltage profile becomes noisy, and the impedance drops to lower values for a further 6 hours until reaching 22 hours. During this period of the experiment, the drop in impedance magnitude indicates the onset of soft shorting in the cell. After 22 hours, the voltage and the impedance dropped to 0 V, which is characteristic of a permanent short circuit in the cell.

A quantification of the total-metal peak signal (from 220–310 ppm) in the *operando*^7^Li NMR spectra shows a gradual increase in intensity, consistent with the formation of Li microstructures (Fig. 10B). No distinct microstructure peak was, however, observed, indicating that dense microstructures are present. The increase in intensity is continuous until a drop in the voltage and impedance after 16 hours. At this point, the total intensity stays constant, which indicates that no further plating on the electrode is occurring due to the formation of a soft short circuit between the electrodes – but one that is more stable (*i.e.*, capable of carrying more current) than that seen in the absence of oxygen. After 22 hours, (*i.e.*, when the voltage and the impedance drop to 0) the total intensity decreases steadily, decreasing by *ca.* 5% between 22 hours and the end of the experiment (45 hours). This is explained by chemical corrosion of the microstructures/short circuits.

The effect of O_2_ on Li-metal plating in TEGDME is clearly detectable by means of *operando* NMR. Without O_2_, the shift of the microstructure peak is higher, indicating that the microstructures are growing away from the bulk-metal surface and forming more dendritic structures, consistent with the SEM images (Fig. 8). In addition, the voltage traces are noisier without O_2_, indicative of more unstable SEIs.^27,28^ The NMR experiments clearly show that the pronounced additional noise seen without O_2_ at approximately 22 hours is associated with type A shorting. There is some recovery between approximately 29–45 hours as the Li continues to plate, albeit at a slower rate, indicating mixed electronic–ionic conduction. Soft short circuits are detected under an O_2_ atmosphere, but these are not recoverable and are not associated with Li plating – *i.e.*, electronic conduction dominates. These differences suggest that the detected soft short circuits in the experiments are based on different degradation mechanisms.

#### 2.2.2 Li -metal plating in DMSO

**Fig. 11:**
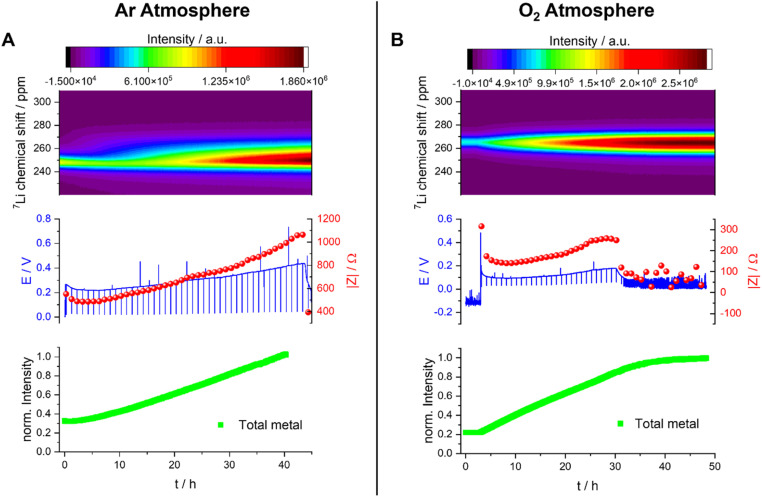
*Operando* NMR spectroscopic measurements of Li-metal plating in symmetrical cells with 1 M LiTFSI in DMSO under Ar (A) and O_2_ (B) atmospheres. *Operando* spectra of the metal between 220 and 310 ppm (top) with the corresponding voltage profile and the impedance magnitude |*Z*| at 9 Hz (middle) and normalised intensity of the metal signal (bottom).

Before plating in DMSO, the Li-metal peak shows a Knight shift of *ca.* 245–265 ppm. The variation in the shift of the bulk metal between experiments (and specifically here with and without O_2_) is tentatively assigned to the BMS effect due to orientation differences in the metal electrodes towards the static magnetic field, and different alignments of the two cells.^29^ When applying a constant current of 1 mA cm^−2^ under Ar, the Li-metal peak becomes broader and asymmetric and increases in intensity (Fig. 11A). This can be explained by the formation of a higher surface area due to the formation of microstructural Li on the surface of the bulk metal. The voltage profile of this experiment shows a smooth plating with an overpotential that grows steadily from around 0.2 to 0.4 V. The measured impedance shows a steady increase during the plating: while the surface area growth typically decreases the impedance, the SEI thickening would increase it. Here, the SEI resistance growth overpowers the resistance decrease due to SA growth. After 45 hours, the voltage and the impedance drops suddenly, indicating either a hard short circuit or a type B soft short circuit.

The quantification of the total Li-metal signal of the cell shows a nearly linear increase in the total-metal peak until the end of the experiment (Fig. 11A), consistent with the increase in the surface area of the metal electrode due to the formation of Li microstructures. The separation in the shifts of the microstructures and the bulk metal is too small to allow deconvolution of the two signals, this small separation indicating that the formed Li microstructures are dense. This is consistent with the SEM, which shows a flake-like morphology of the plated Li (Fig. 8).

The overpotential is lower (around 0.15 V) when measuring the Li-metal plating in DMSO under O_2_, and it increases only slightly during the first 30 hours of the experiment (Fig. 11B). During this time period, the intensity of the metal peak increases and broadens, both being consistent with continuous Li plating. The Li-metal signal is more symmetric than the signal seen in the absence of O_2_, which is consistent with a smoother deposition of Li metal. After 30 hours, the voltage and the impedance drop to lower values, but not to zero, and become noisier, both being indicative of a degradation mechanism based on soft shorts. At this point, the Li-metal signal no longer grows, which can be correlated to an inhibition of further metal plating due to soft short circuits in the cell. These soft short circuits are stable and remain until the end of the experiment.

The Nyquist plot of the impedance measurements in the first 30 hours (Fig. S7B†) consists of at least one semi-circle with a maximum of around 500 Hz. The Nyquist plot of the measurement taken after unidirectional plating of 30 mA h cm^−2^ has a typical shape for soft-shorted cells without distinguishable semi-circles (Fig. S7B†).

Under both Ar and O_2_, soft short circuits form after unidirectional plating for more than 30 hours (*i.e.*, plating at 30 mA h cm^−2^). However, the relatively low overpotential and more reversible soft shorts seen under O_2_ are closer to the type A shorts seen for TEGDME. The single, abrupt, hard short circuit under the Ar atmosphere (type B) again supports the existence of alternative soft-short formation mechanisms.

## 3 Summary and conclusion

We have reviewed some of the experimental methods to study the function and failure modes in lithium–air batteries, with a strong focus on the NMR experiments developed within our group. Oxygen is a paramagnetic molecule and its presence in the cell can affect the NMR parameters of the observed NMR nuclei (shifts, relaxation times), but this can in turn be used to quantify its concentration and diffusivity and to probe its interaction with electrolyte species. The reaction and degradation products, including the formation of Li-metal dendrites on the anode, can all be followed by both solid- and solution-state NMR spectroscopy, allowing a variety of degradation mechanisms to be studied. In particular, the ability to enrich oxygen with the NMR-active nucleus ^17^O means that the fate of oxygen in the cell can be tracked. The mechanistic changes caused by addition of redox mediators could be studied by using both EPR and NMR: EPR complements solution NMR particularly well in such studies, because the radicals often lead to paramagnetic relaxation and hence NMR peak broadening, along with large shifts in the resonances, making species hard to detect and/or identify. Thus, if such radicals can be detected *via* EPR, the redox mediator can be followed in both its oxidised and reduced state. One can also use EPR more broadly to study lithium–air batteries; for example, it can be used in combination with spin traps to monitor singlet-oxygen production.^30^

Finally, we have demonstrated that soft-short formation occurs in Li symmetric cells with common lithium–air electrolytes, the results indicating that for a high-rate LAB, the lithium anode may become limiting. Results exploring these phenomena in O_2_ atmospheres suggests that a more favourable, albeit more resistive, SEI is formed in the presence of O_2_; this SEI is able to support more stable Li plating with a moderate increase in overpotential. Although it is encouraging that the effect of soft shorts is less under the O_2_ atmosphere, further studies of anode degradation in full Li–air (rather than Li–O_2_) cells, with redox mediator-containing electrolytes, and under more practical cycling profiles need to be conducted to mitigate the impact of these shorts. The approaches outlined here can similarly be used to study soft shorts and degradation more widely in a wider range of systems, including Na-ion and Na–air batteries.

## 4 Methodology

### 4.1 Materials

Anhydrous tetraethylene glycol dimethyl ether (TEGDME, Sigma-Aldrich, 99.0% pure), and dimethylsulfoxide (DMSO, Sigma-Aldrich, 99.9% pure) were purified in contact with metallic sodium for three days, distilled immediately afterwards and stored in contact with 4 Å molecular sieves. Lithium bis(trifluoromethane sulfonyl)imide (LiTFSI, Sigma-Aldrich, 99.99% pure) was dried at 150 °C in a vacuum oven and transferred to an Ar-filled glovebox without contact with the atmosphere. Lithium chips (PI-KEM) were used as received. All chemicals were stored in an argon-filled MBRAUN glove box with oxygen content lower than 0.1 ppm, and water content below 2 ppm. All solutions were prepared inside the glove box.

### 4.2 Electrochemical cycling

Symmetric Li–Li coin cells and Swagelok-type cells were used to test galvanostatic cycling and EIS. Swagelok cells with a modified plunger attached to a valve were used to allow oxygen into the cell, as described elsewhere.^4^ A modification of this design to account for the non-porous Li electrode includes the replacement of a Cu for a stainless-steel mesh to give mechanical stability, and the use of small electrodes (5 mm in diameter) to promote fast O_2_ diffusion times from the headspace to the centre of the electrode. Galvanostatic cycling was carried out for two cycles at a current density of 0.2 mA cm^−2^, and then it was doubled every two cycles until it reached 1.6 or 3.2 mA cm^−2^. The potential was recorded along the cycling curve, and galvanostatic impedance spectroscopy (GEIS) was performed every 2–2.5 hours using a current amplitude of 100 μA. The points in time where EIS was measured correspond to different stages in the lithium plating/stripping cycles: EIS was measured at the beginning of each half cycle, and three more times throughout it.

### 4.3 Scanning electron microscopy (SEM)

After electrochemical plating for 3 hours with a current density of 1 mA cm^−2^, the coin cells were transferred into an Ar Glovebox and disassembled. The Li-metal electrode was mounted onto the SEM stage of the transfer module (Kammrath & Weiss, type CT0) and dried under vacuum for 1 hour. The electrodes were not rinsed with a solvent before the to avoid removal of loosely attached lithium microstuctures. The samples were transferred into the SEM chamber using the air-sensitive transfer module under an inert atmosphere (Ar), without being exposed to air. SEM images were acquired with a Tescan MIRA3 FEG-SEM instrument at an acceleration voltage of 5.0 kV.

### 4.4 *Operando* NMR measurements

The *operando* NMR experiments were conducted on a Bruker Avance 300 MHz spectrometer (the Larmor frequency for ^7^Li being 116.6 MHz) using a solenoidal Ag-coated Cu coil. The spectra were recorded using an *in situ* automatic-tuning-and-matching probe (ATM VT X *in situ* WB NMR probe, NMR Service) that allows for an automatic recalibration of the NMR rf-circuit during an *in situ* electrochemistry experiment. The retuning of the rf-circuit becomes essential in order to quantify the NMR signals when the sample conditions are changing during the electrochemistry.^31^ The probe has highly shielded wire connections to the electrochemistry circuit with low-pass filters (5 MHz) attached to the probe, minimizing the interference between the NMR and the electrochemistry circuit. Overall, the *in situ* setup allows for highly reproducible NMR measurements. Single-pulse experiments were used to collect the NMR data, with a recycle delay of 1 s (>5 × *T*_1_) and 256 transients recorded. This resulted in an experimental time of about 4.5 min. The shift of ^7^Li was referenced to 1 M LiCl in water at 0 ppm. The spectra were processed in the Bruker Topspin software using the automatic phase and baseline correction. Further data processing was done in R. The total intensity of the Li-metal peak was integrated over the ^7^Li shift range of 310–220 ppm and normalized to the intensity measured at the end of plating. Due to the use of metallic electrodes in the cell, the skin depth effect must be considered when NMR spectra of lithium cells are recorded. To detect any change in the surface area, the total-metal peak (220 to 310 ppm) was integrated and plotted as “total metal” in this paper. The formed microstructure during the deposition, however, is reported in the literature to be smaller than the skin depth, and therefore, the intensity of the microstructure peak is directly proportional to their mass. The quantification of the metal signals followed the theory developed and described by Bhattacharyya *et al.*^26^

For the *in situ* NMR cell setup in this study, all the cells operate with an applied current density of 1 mA cm^−2^. The calculated limiting current density is ∼7 mA cm^−2^.^24^ Thus, the galvanostatic experiments performed in this work are in the low current density regime.^32^ A capsule cell (NMR service)^33^ was used for all *in situ* NMR experiments under Ar, with a modified cell^11^ being used for experiments under oxygen, both cells being made out of PEEK. Working electrodes consisted of Li-metal foil. The amount of electrolyte added to each cell was 100–150 μL.

## Conflicts of interest

CPG is a co-founder and a shareholder in a fast-charging battery company (Nyobolt).

## Supplementary Material

FD-248-D3FD00154G-s001
