# Supplementary material for: Spiers Memorial Lecture: Lithium air batteries – tracking function and failure
Source: Faraday Discuss. 2023 Dec 4;248:9–28. doi: 10.1039/d3fd00154g (PMC10823487; doi:10.1039/d3fd00154g)
Supplement: FD-248-D3FD00154G-s001 [file FD-248-D3FD00154G-s001.pdf]

Electronic Supplementary Material (ESI) for Faraday Discussions.
This journal is © The Royal Society of Chemistry 2023

# ESI for Lithium Air Batteries – Tracking Function and Failure

Jana B. Fritzke[a], James H. J. Ellison[a], Laurence Brazel[a], Gabriela Horwitz[a], Svetlana Menkin[a,**]and Clare P. Grey*[,a]

## Galvanostatic cycling of Li-Li cells

Voltage traces of galvanostatic cycling of Li-Li symmetric Swagelok cells with different atmospheres (Figures S1-S4) and coin cells (Figures S5 and S6) are shown below with 1M LiTFSI in DMSO and 1M LiTFSI in TEGDME electrolytes. Comparing Swagelok cells cycled with DMSO-based electrolytes (Figures S1 and S2) and TEGDME-based electrolytes (Figures S3 and S4), it can be seen that the DMSO-based electrolytes form soft shorts at lower current densities compared to TEGDME-based electrolytes with the same atmosphere. Soft shorting is evidenced by the sudden overpotential drop, impedance drop and the rectangular voltage traces. It can be seen, however, that the cells with TEGDME-based electrolyte had noisier voltage profiles, potentially due to dead lithium accumulation or softer shorts (i.e., the fuse that connects the electrodes is weaker and, as a result, these shorts are most recoverable).

Comparing the Ar atmosphere (Figure S1 for DMSO-based electrolyte, S3 for TEGDME-based electrolyte) to $O_2$ atmosphere (Figure S2 for DMSO-based electrolyte, S4 for TEGDME-based electrolyte), it can be seen that for both DMSO- and TEGDME-based electrolyte the presence of $O_2$ increased the critical current density (i.e., the cell failed at higher current density).

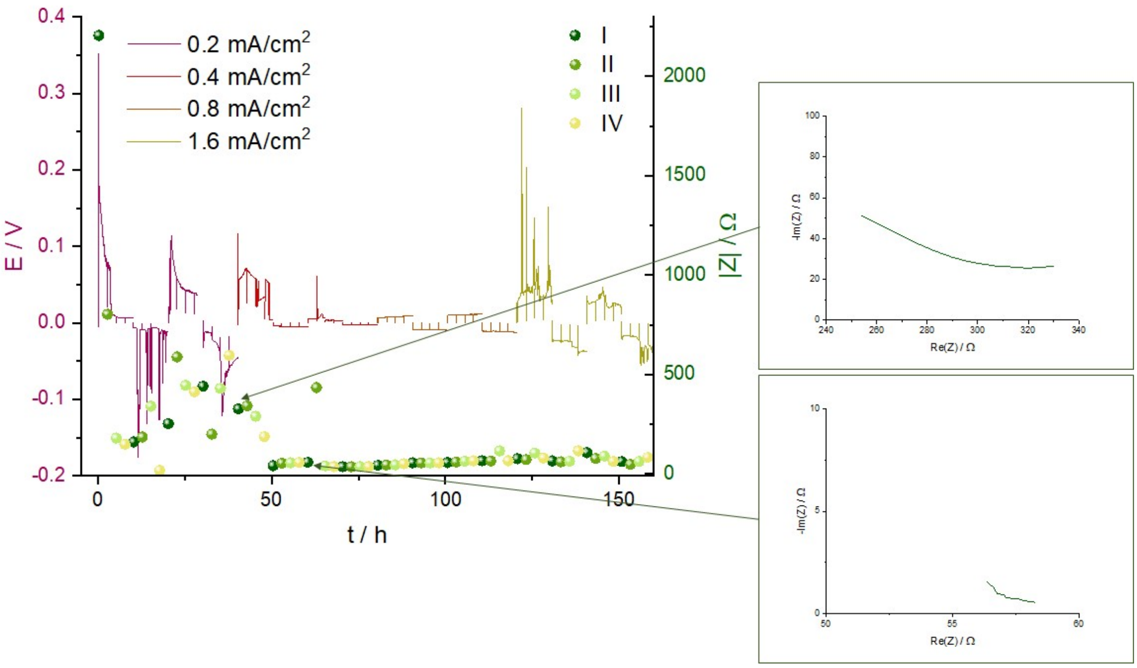


**Figure S1:** voltage trace of galvanostatic cycling for Li-Li symmetric Swagelok cells in 1M LiTFSI electrolyte in DMSO in Ar atmosphere and the corresponding impedance magnitude |Z| at 9 Hz. Inserts are Nyquist plots before and after soft shorting occurs.

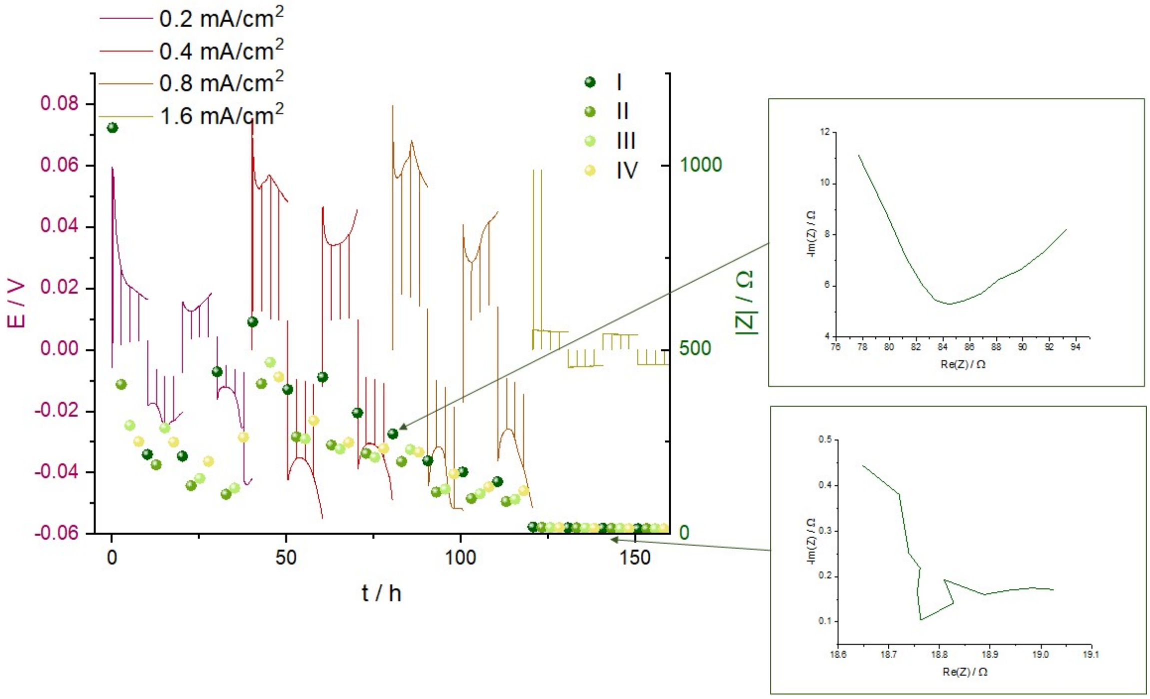


**Figure S2:** voltage trace of galvanostatic cycling for Li-Li symmetric Swagelok cells 1M LiTFSI electrolyte in DMSO in $O_2$ atmosphere and the corresponding impedance magnitude |Z| at 9 Hz. Inserts are Nyquist plots before and after soft shorting occurs.

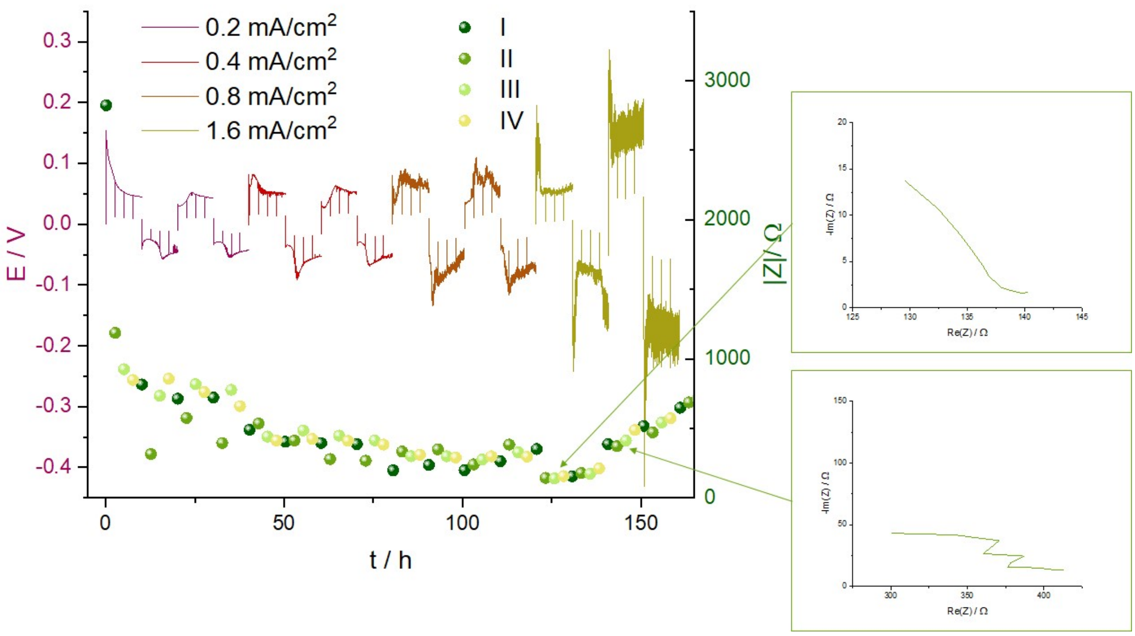


**Figure S3**: voltage trace of galvanostatic cycling for Li-Li symmetric Swagelok cells 1M LiTFSI electrolyte in TEGDME in Ar atmosphere and the corresponding impedance magnitude |Z| at 9 Hz. Inserts are Nyquist plots before and after soft shorting occurs.

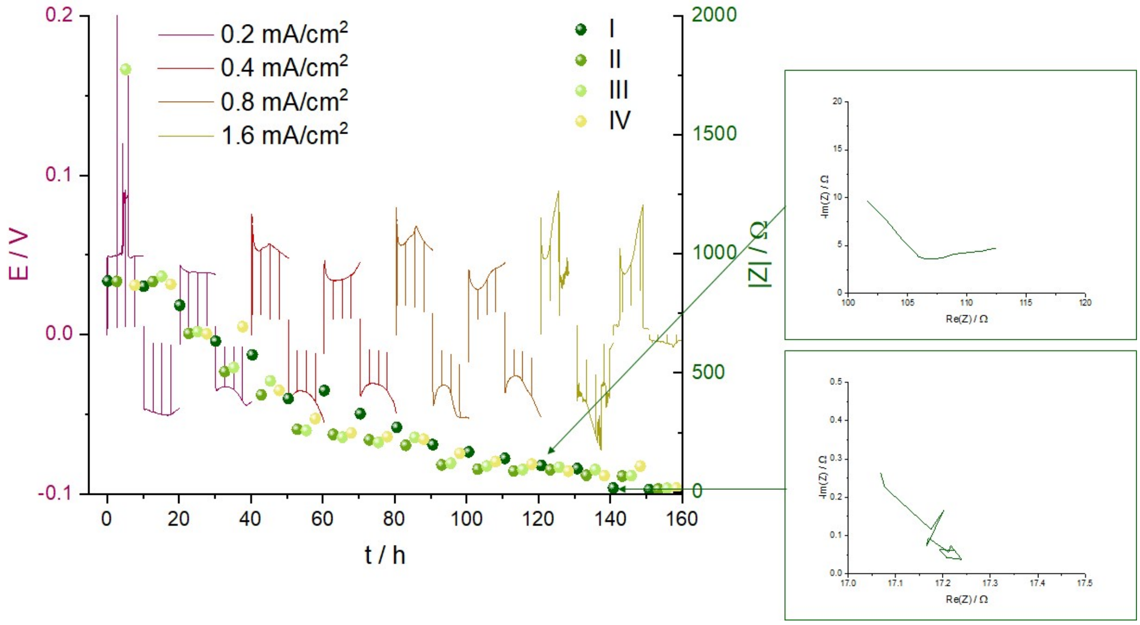


**Figure S4**: voltage trace of galvanostatic cycling for Li-Li symmetric Swagelok cells 1M LiTFSI electrolyte in TEGDME in $O_2$ atmosphere and the corresponding impedance magnitude |Z| at 9 Hz. Inserts are Nyquist plots before and after soft shorting occurs.

Figures S5 and S6 show voltage traces of Li-Li symmetric coin cells prepared in an Ar atmosphere with TEGDME- and DMSO-based electrolytes, respectively. As seen with the Swagelok cells above, the DMSO-based electrolyte leads to soft shorts at a lower current density than the TEGDME-based electrolyte, the latter of which did not appear to be soft short during the experiment. The noisy voltage profile may indicate a significant build-up of dead lithium or more transient shorts.

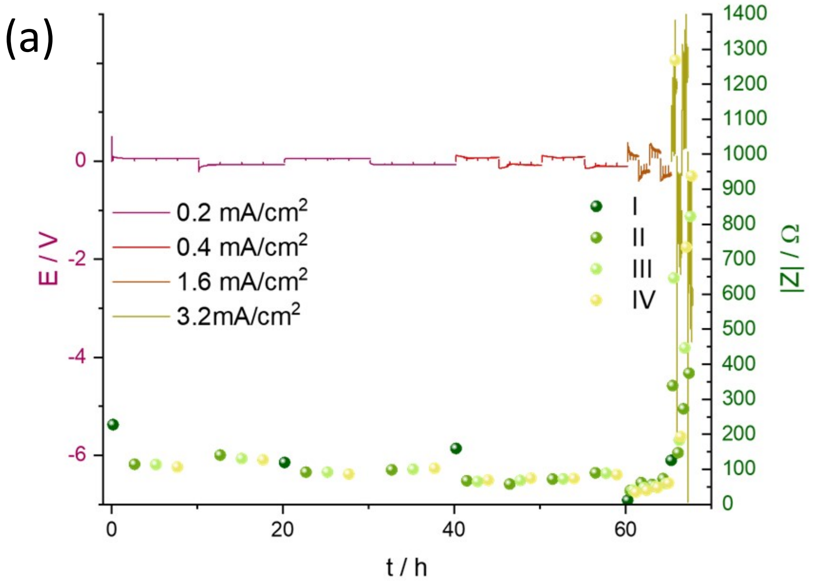

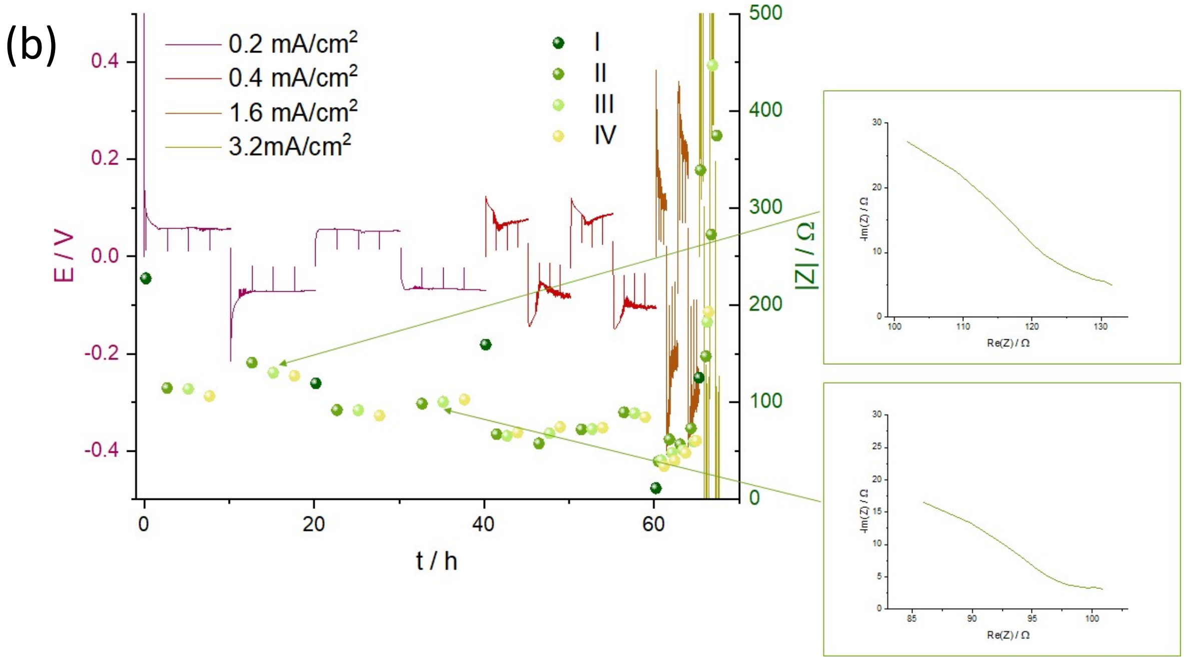


**Figure S5**: voltage trace of galvanostatic cycling for Li-Li symmetric coin cells 1M LiTFSI electrolyte in TEGDME prepared in Ar atmosphere and the corresponding impedance magnitude |Z| at 9 Hz (A) full scale (B) rescaled. Inserts are Nyquist plots with no clear evidence of soft shorting occurring.

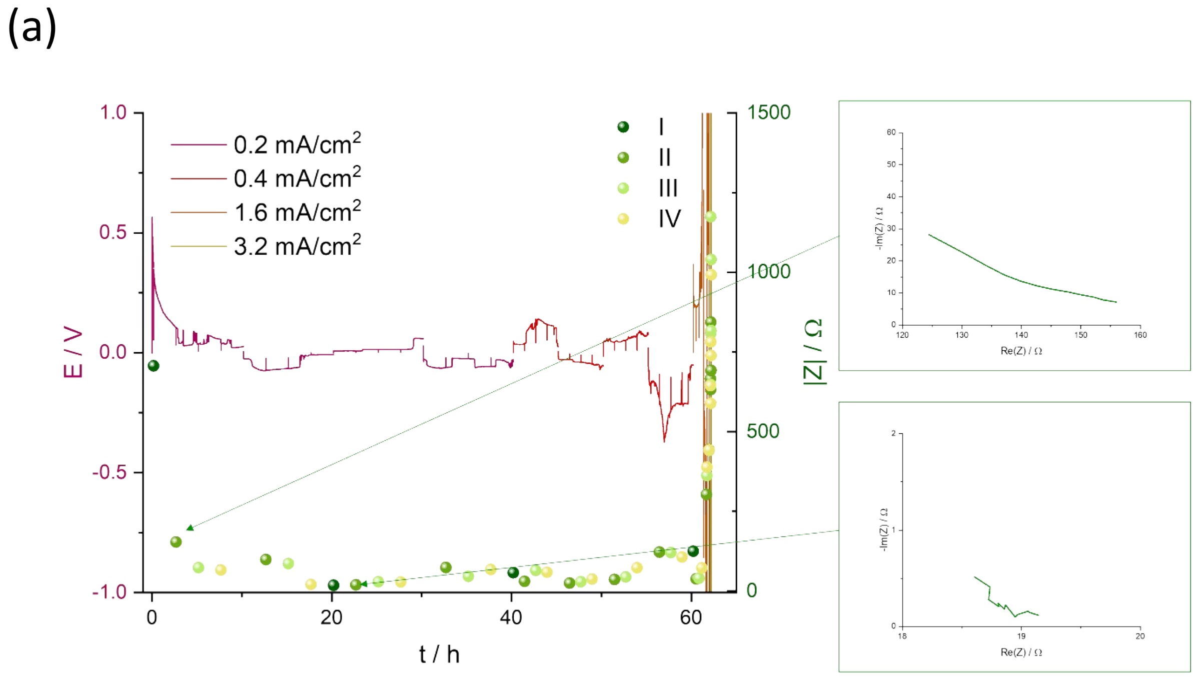

(b)

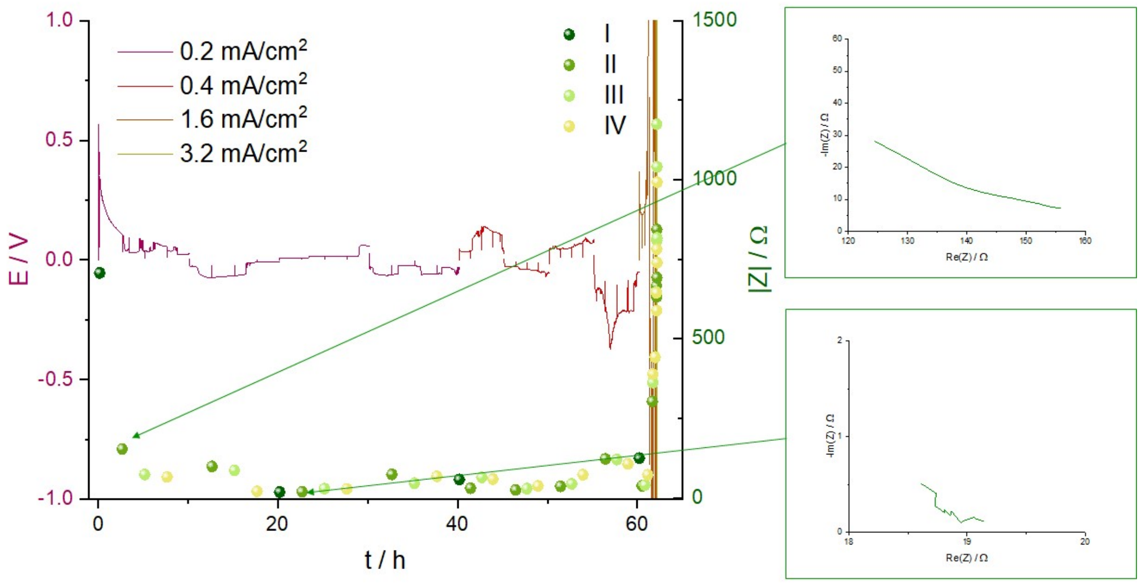


**Figure S6**: voltage trace of galvanostatic cycling for Li-Li symmetric coin cells 1M LiTFSI electrolyte in DMSO prepared in Ar atmosphere and the corresponding impedance magnitude |Z| at 9 Hz (a) full scale (b) rescaled. Inserts are Nyquist plots before and after soft shorting occurs.

## Operando NMR of Li-Li symmetric cells

Figure S7 shows the galvanostatic voltage trace and corresponding impedance values (A) and Nyquist plots (B) of the operando NMR Li-Li symmetric cell with a 1M LiTFSI in a DMSO electrolyte in an $O_2$ atmosphere. The cell soft shorted at 30 hours, corresponding to a sudden drop in overpotential and impedance. In Figure S7A, the total intensity of the Li metal peak can be seen to gradually stop increasing after the soft short appeared, indicating no more metal plating is occurring. The shape of the Nyquist plot supports the formation of soft shorts approximately at 30 h. An increase was measured after the initial impedance drop during the first ten hours of plating, potentially due to SEI accumulation. Then, after 35 hours, there was a sudden drop, and the Nyquist plot shape transitioned into the shape typical for a short-circuited cell.

Figure S8 shows the galvanostatic voltage trace and corresponding impedance values (A) and Nyquist plots (B) of the *operando* NMR Li-Li symmetric cell with a 1M LiTFSI in TEGDME electrolyte in an $O_2$ atmosphere. After 16 hours, the cell soft shorted, and the potential and impedance dropped to 0 after 22 hours, indicating a hard short. During the rest of the experiment, the total metal signal slowly decreased, indicating the corrosion of metal microstructure while no further plating occurred.

The Nyquist plot of the first impedance measurement (Figure S8B, orange) consists of at least one semi-circle with a maximum of around 500 Hz. The next two highlighted impedance measurements (Figure S8B, green and blue) taken after unidirectional plating of 10 and 15 mAh cm $^{-2}$, respectively, consist of at least one semi-circle with a maximum frequency in the range between 250 and 100 Hz (Figure S8B). We attribute this change to the SEI thickening.

Figure S9 shows the galvanostatic voltage trace and corresponding impedance values (A) and Nyquist plots (B) of the *operando* NMR Li-Li symmetric cell with a 1M LiTFSI in TEGDME electrolyte in an Ar atmosphere. Although there is no clear indication of a soft short until approximately 20 hours, the voltage trace is very noisy (consistently with the corresponding Swagelok cell; see Figure S3). The shape of the Nyquist plot is typical for a short-circuited cell from the first measurement, suggesting that soft shorts were formed even at the first nucleation.

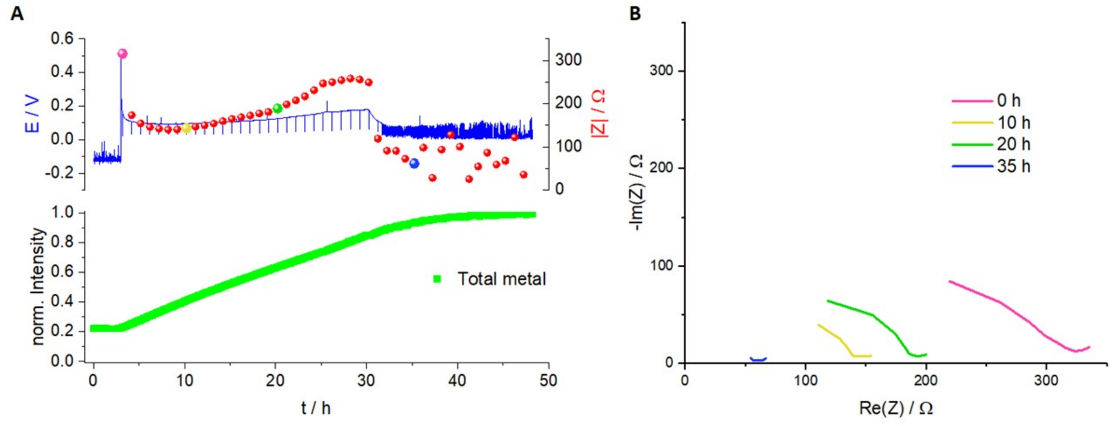


**Figure S7:** Operando NMR spectroscopic measurements of Li metal plating at 1mA cm$^{-2}$ in symmetrical Li cell with 1 M LiTFSI in DMSO under $O_2$ atmosphere (A) and the Nyquist plots during plating (B).

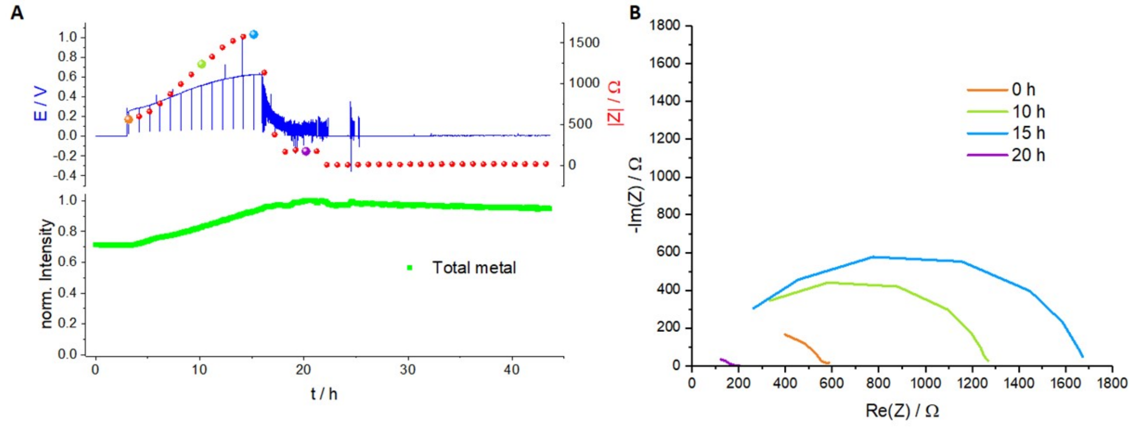


**Figure S8:** Operando NMR spectroscopic measurements of Li metal plating at 1mA cm$^{-2}$ in symmetrical Li cell with 1 M LiTFSI in TEGDME under $O_2$ atmosphere (A) and the Nyquist plots during plating (B).

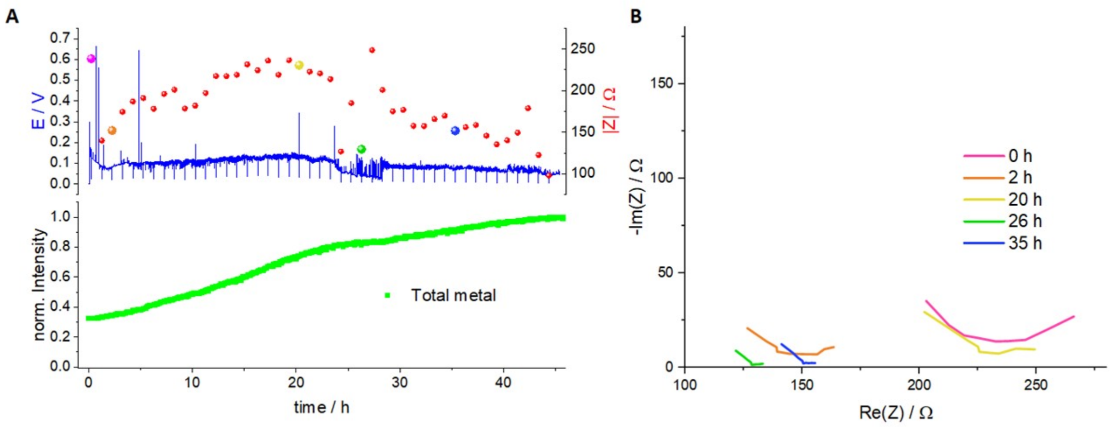


**Figure S9:** Operando NMR spectroscopic measurements of Li metal plating at 1mA cm$^{-2}$ in symmetrical Li cell with 1 M LiTFSI in TEGDME under Ar atmosphere (A) and the Nyquist plots during plating (B).
